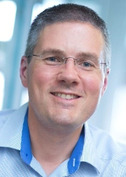# Molecular Vanadium Oxides for Energy Conversion and Energy Storage: Current Trends and Emerging Opportunities

**DOI:** 10.1002/anie.202010577

**Published:** 2020-12-17

**Authors:** Montaha Anjass, Grace A. Lowe, Carsten Streb

**Affiliations:** ^1^ Institute of Inorganic Chemistry I Ulm University Albert-Einstein-Allee 11 89081 Ulm Germany; ^2^ Helmholtz Institute Ulm Helmholtzstrasse 12 89081 Ulm Germany

**Keywords:** materials design, metal oxides, polyoxometalates, polyoxovanadates, self-assembly

## Abstract

Molecular vanadium oxides, or polyoxovanadates (POVs), have recently emerged as a new class of molecular energy conversion/storage materials, which combine diverse, chemically tunable redox behavior and reversible multielectron storage capabilities. This Review explores current challenges, major breakthroughs, and future opportunities in the use of POVs for energy conversion and storage. The reactivity, advantages, and limitations of POVs are explored, with a focus on their use in lithium and post‐lithium‐ion batteries, redox‐flow batteries, and light‐driven energy conversion. Finally, emerging themes and new research directions are critically assessed to provide inspiration for how this promising materials class can advance research in sustainable energy technologies.

## Graphical abstract

This Review critically discusses recent breakthroughs and future challenges in research on polyoxovanadate energy materials. The use of polyoxovanadates in batteries, redox‐flow batteries, light‐driven catalysis, and electrocatalysis is described together with an outlook on emerging themes and areas of future application.

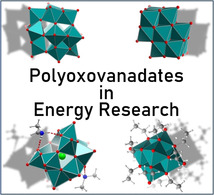

## 1 Introduction

Our way of harvesting and storing energy is beginning to change on a global scale. The transition from traditional fossil‐fuel‐based systems to carbon‐neutral and more sustainable schemes is underway.^[1]^ With this transition comes the need for new directions in energy materials research to access advanced compounds for energy conversion, transfer, and storage. In addition, long‐term stability, economic viability, and sustainability will become central design criteria.[^2^, ^3^] In particular, redox‐active materials are essential for electrochemical or photochemical energy conversion and storage, including solar fuels, photovoltaics, electrocatalysis, and batteries. Metal oxides have become established as a chemically suitable and economically viable materials class for energy technologies such as batteries,^[4]^ water electrolysis,^[5]^ fuel cells,^[6]^ and small‐molecule (e.g. H_2_O, CO_2_, N_2_) activation.^[7]^

Over the last decade, molecular metal oxides (polyoxometalates, POMs) have become a focal point in energy materials research. POMs combine the fundamental chemical properties of solid‐state metal oxides with the structural precision and chemical tunability inherent to molecular systems.[^8^, ^9^, ^10^] As such, they have shown outstanding performance in challenging energy‐related applications, including water oxidation,[^11^, ^12^] hydrogen evolution,[^13^, ^14^] and batteries.[^15^, ^16^] Traditionally, most POM research was focused on W‐ and Mo‐based systems, that is, polyoxotungstates and polyoxomolybdates, with a focus on derivatives of the Keggin [XM_12_O_40_]^*n*−^ and Wells‐Dawson [X_2_M_18_O_62_]^*m*−^ anions (X=P, Si, etc. and M=Mo, W).[^17^, ^18^, ^19^, ^20^]

**Figure 1:**
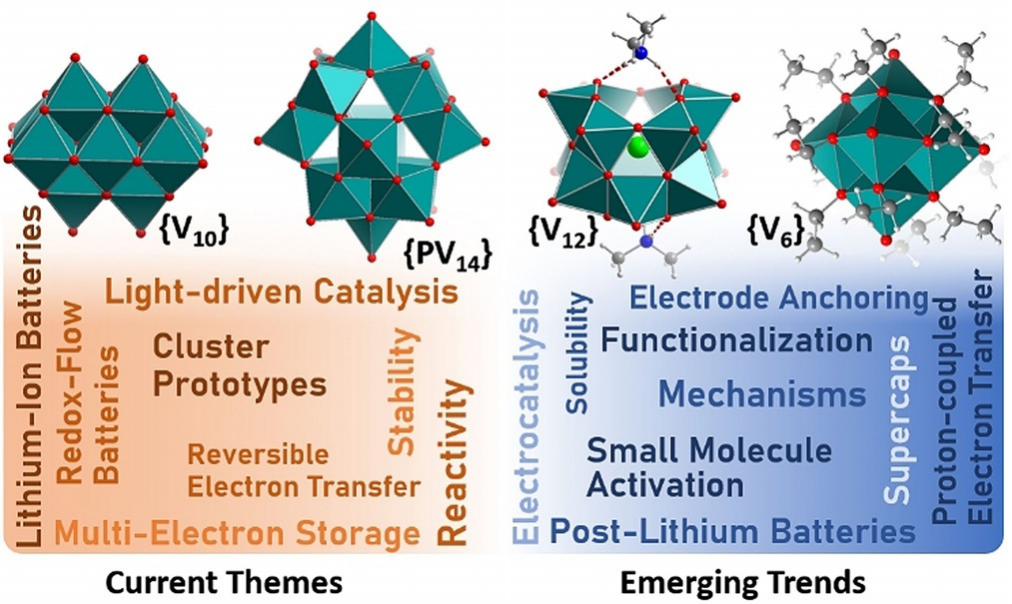
Top: Illustration of the prototype clusters typically used in current POV energy research (**{V_10_}**=[V_10_O_28_]^6−^; **{PV_14_}**=[PV_14_O_42_]^9−^; **{V_12_}**=[V_12_O_32_Cl]^5−^; **{V_6_}**=[V_6_O_7_(OEt)_12_]). Bottom: Keywords describing current and future themes in POV energy research.

Although tremendous progress was made on tungstate and molybdate chemistry in the 1990s and early 2000s, molecular vanadium oxides, or polyoxovanadates (POVs; Figure 1),[^21^, ^22^, ^23^] have only received widespread attention in the area of energy research over the last decade. POVs feature multiple accessible oxidation states (mainly based on the V^V^/V^IV^ and more rarely V^IV^/V^III^ redox couples), resulting in rich redox behavior.^[24]^ In addition, they have a significantly lower atomic weight than other POM families (V: 50.94 g mol^−1^; Mo: 95.95 g mol^−1^; W: 183.84 g mol^−1^), which allows higher gravimetric energy densities and makes POVs relevant for battery applications.^[25]^ Furthermore, vanadium is obtained as a by‐product of steel manufacturing and is, therefore, available on an industrial scale.^[26]^ In addition, POVs are easily disassembled in alkaline media, which could open technologically feasible routes to POV recycling and thus leading to a more stable vanadium supply chain. However, as described in the following, the development of POVs faces significant challenges because of their complex formationand functionalization combined with their (perceived) lower stability compared with other POM classes.[^21^, ^23^, ^27^]

The functionalization of molybdates and tungstates with redox‐active heterometals has been widely used to tune their redox behavior and resulting reactivity for applications such as, water oxidation,[^11^, ^12^] hydrogen evolution,^[16]^ photooxidation chemistry,^[28]^ and battery applications.^[29]^ However, until recently, the functionalization of POVs with metals was mainly used for structural stabilization,[^27^, ^30^, ^31^, ^32^] precursor synthesis,[^33^, ^34^] or to access new cluster topologies.[^21^, ^23^, ^27^] Over recent years, however, POV chemistry has moved from exploring structures to functions, which has enabled ground‐breaking research in many areas of energy conversion and storage. In this Review, we will showcase the recent developments in the redox tuning of POVs. We highlight the unique advantages of POVs that can be exploited in energy research and identify areas which—from the authors’ point of view—offer new possibilities for fundamental and application‐driven research over the coming years.

## 2 Fundamentals of Polyoxovanadate Chemistry

The principal reactivity of POVs has been described in the literature.[^21^, ^22^, ^23^, ^27^, ^35^, ^36^] Therefore, only a brief overview of their most important features is given here: POVs self‐assemble in aqueous or organic solvents,[^21^, ^22^] sometimes involving internal templates such as transition metals,[^27^, ^37^] anions (halides, pseudohalides, oxoanions),[^21^, ^23^, ^27^] or, very rarely, neutral organic compounds.^[38]^ The functionalization of POVs with metal cations,[^23^, ^27^] semimetals,^[36]^ or organic groups, for example, alkoxides,^[39]^ gives access to derivatives that feature new structures and reactivities. In contrast to molybdates and tungstates, which are typically based on [MoO_6_]/[WO_6_] octahedra as fundamental building units, POVs show more structural flexibility and feature tetrahedral [VO_4_], square‐pyramidal [VO_5_], and octahedral [VO_6_] coordination modes.[^21^, ^27^] However, this coordinative versatility can also facilitate structural rearrangements, which needs to be considered when deploying POVs in energy applications.[^27^, ^40^, ^41^, ^42^, ^43^] In addition, POVs are often described as being “less stable” than tungstates and molybdates, which is most likely due to a combination of their structural flexibility, complex protonation chemistry, and high redox activity.[^21^, ^23^, ^27^]

These challenges also offer opportunities, particularly for energy materials research: key features which render POVs interesting for energy conversion and storage are their capability to reversibly store and transfer multiple electrons, based on the accessibility of the V^V^/V^IV^ and—more rarely—the V^IV^/V^III^ redox couples.^[24]^ Despite its importance for batteries, supercapacitors, and electrocatalysis, multielectron storage in POVs is still underexplored.[^44^, ^45^] In addition, the related area of proton‐coupled electron transfer in POVs requires urgent attention,^[46]^ as it could facilitate complex multielectron transfers (e.g. water oxidation, hydrogen evolution, CO_2_ reduction) to overcome activation barriers or tune redox potentials.^[47]^ Another unique feature of POVs is their smaller HOMO–LUMO gap compared with those of Mo‐ or W‐based POMs, so that POVs can act as efficient visible‐light‐driven photoredox catalysts.^[48]^ In the following sections, we will discuss recent developments in the use of POVs in lithium‐ion and post‐lithium‐ion batteries, redox‐flow batteries, and photochemistry. We will describe unique features as well as current limitations of POVs and provide an outlook on future scenarios for POV‐based energy research.

## 3 POVs in Batteries

**Figure 2:**
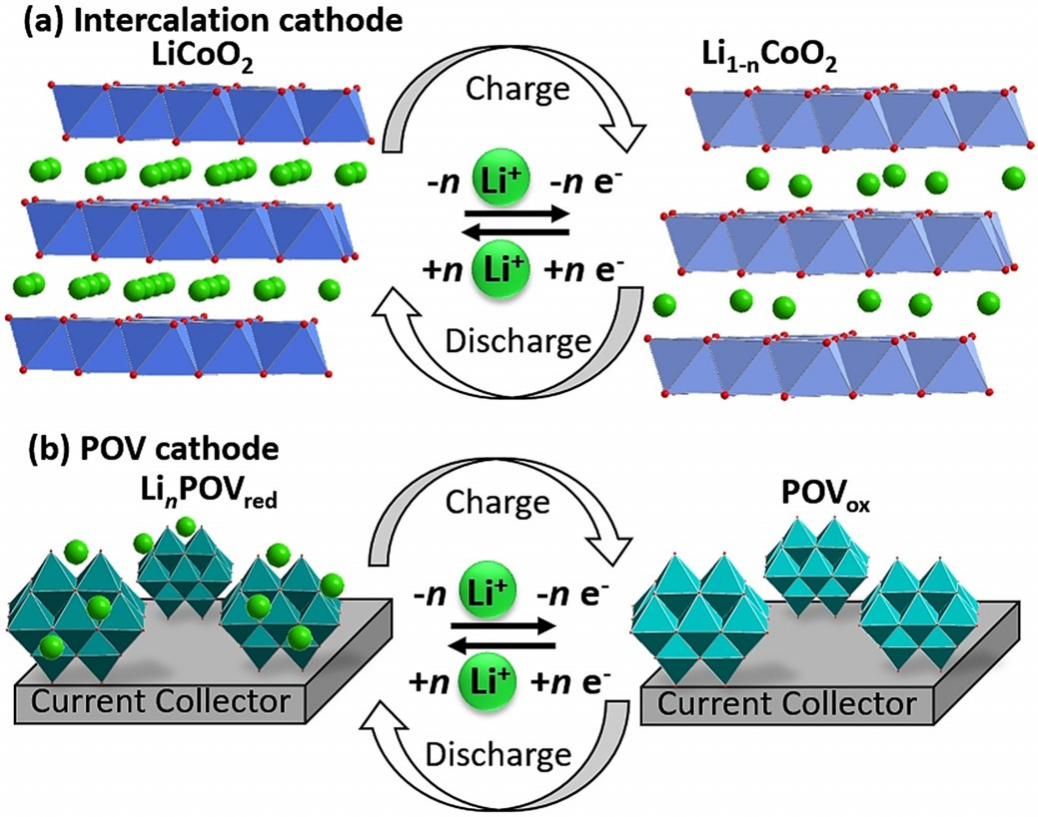
Schematic illustration of the charge–discharge mechanism of a) classical LIB intercalation compounds (e.g. LiCoO_2_) and b) POV‐based battery electrodes (exemplified here by **{V_10_}**).

Rechargeable batteries are key electrochemical energy storage technologies where stored chemical energy is converted into electricity.^[49]^ Currently, lithium‐ion batteries (LIBs) are amongst the most successful electrochemical energy storage technologies, as they offer high voltages and, thus, high energy densities. However, the currently used electrode materials appear not to be sustainable in the medium and long term because of the limited availability of several of the elements used (e.g. Li, Co).^[50]^ Furthermore, LIBs typically rely on so‐called intercalation compounds (often metal oxides), where the cycling stability is limited by the mechanical degradation of the structure during the lithium intercalation/extraction (Figure 2).^[51]^ To create more‐stable battery electrodes, a new class of battery electrode materials is required which combines high energy density and stability with chemical tunability and economic viability.

POMs have inspired ground‐breaking research on battery materials as they can bridge the gap between molecular designer materials and technologically important solid‐state metal oxides. Whereas pioneering studies of POMs as battery components were focused on polyoxomolybdates,[^16^, ^52^, ^53^] recent studies have reported breakthroughs in the use of POVs as active components for battery electrodes. POVs are the lightest class of POMs available and they, therefore, offer higher gravimetric energy densities, which is important for e‐mobility applications. In addition, POVs can, in principle, undergo multiple redox processes per V center (POVs containing V^V^, V^IV^, and more rarely V^III^ have been reported),^[24]^ which would further increase their electron‐storage capacity.^[54]^ These benefits combined with the economic viability of vanadium has led to fast‐paced progress in the exploration of POV‐based batteries.^[55]^

To date, most studies have been focused on the POV prototype decavanadate, which is typically synthesized under aqueous conditions and isolated as a hydrated salt, that is, M_6_[V_10_O_28_]⋅*x* H_2_O (M=Li or Na; *x=*9–16). As a consequence of this synthetic approach, the crystal lattice typically contains water, which needs to be thermally removed, as otherwise this would lead to “gassing” (O_2_ and H_2_ evolution) under typical LIB operation conditions.^[56]^ For this reason, previous studies used thermally dehydrated Li_6_[V_10_O_28_] as a LIB cathode material, which led to initial discharge capacities of about 130 mAh g^−1^ at a current density of 0.2 mA cm^−2^ and potentials between 2.0 and 4.2 V vs. Li^+^/Li.^[57]^ Subsequent studies investigated the performance of the component after thermal treatment at higher temperatures (450 °C). The samples showed high initial specific capacities (ca. 400 mAh g^−1^) but low cycling stability.^[58]^ In contrast, samples treated at 600 °C showed lower initial specific capacities (ca. 200 mAh g^−1^) but high cycling stability.^[58]^ Subsequent studies used the thermally dehydrated decavanadate salt Na_6_[V_10_O_28_] as the cathode active material in LIBs. By using in situ X‐ray absorption near‐edge spectroscopic (XANES) studies, the authors showed that all ten V^5+^ ions can be reversibly reduced to V^4+^ in a potential range of 4–1.75 V vs. Li^+^/Li.^[59]^ The decavanadate was also used as a starting material for the fabrication of POV‐based electrodes for sodium‐ion batteries (NIBs), and Na_6_[V_10_O_28_] was reported as an anode material with a reversible capacity of about 280 mAh g^−1^, an average discharge potential of 0.4 V vs. Na^+^/Na, and high cycling stability.^[55]^ In most of these studies, the removal of lattice water involved thermal treatment at high temperatures. However, POVs are known to easily convert into solid‐state vanadium oxides,[^60^, ^61^] and the decavanadate cluster is particularly susceptible to undergo thermally induced structural rearrangements; the dehydration of lithium decavanadate Li_6_[V_10_O_28_]⋅16 H_2_O leads to the formation of two solid‐state oxides, LiVO_3_ and LiV_3_O_8_, even at moderate temperatures of about 120 °C.^[62]^ Consequently, most studies of decavanadates as molecular electrode components reported to date were in fact analyzing the performance of nano‐ or microstructured solid‐state lithium vanadium oxides.^[62]^

In addition to studies using the decavanadate prototype, other common POVs such as K_5.72_H_3.28_[PV_14_O_42_],^[64]^ K_7_[NiV_13_O_38_],^[65]^ K_7_[MnV_13_O_38_],^[66]^ and the carbonate‐templated Li_7_[V_15_O_36_(CO_3_)][^67^, ^68^] have also been used as NIB and LIB cathode materials and showed promising performance. In addition, Dong, Cronin, and co‐workers designed symmetric LIBs that employed Li_7_[V_15_O_36_(CO_3_)] as the active anode and cathode material. The system combined battery‐like energy density (125 Wh kg^−1^) and supercapacitor‐like power density (51.5 kW kg^−1^ at 100 A g^−1^) and could serve as a model to bridge these two technologies.^[68]^ However, as discussed for the decavanadate, questions remain on the actual structure of the active materials and their chemical evolution under battery cycling conditions.^[61]^

**Figure 3:**
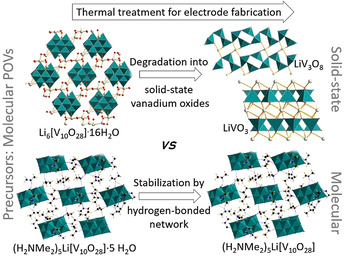
Top: illustration of the thermal conversion of molecular decavanadate into solid‐state vanadium oxides during dehydration and electrode fabrication.^[62]^ Bottom: Stabilization of decavanadate under electrode fabrication conditions by electrostatic and hydrogen bonding using dimethylammonium cations.^[63]^

To explore the performance of truly molecular POV battery electrodes, Anjass, Streb, and co‐workers proposed the use of supramolecular crystal engineering for POV stabilization.^[63]^ To this end, dimethylammonium cations were used for electrostatic and hydrogen‐bonding stabilization of the decavanadate cluster in the crystal lattice. This prevented thermal degradation of the POV into solid‐state oxides and led to the retention of the molecular structure of decavanadate in the resulting LIB electrodes (Figure 3). Initial tests of these cathodes in LIBs showed specific capacities up to 290 mA h g^−1^ in a voltage range between 1.2 and 3.4 V vs. Li^+^/Li, at a current density of 50 mA g^−1^. The viability of this POV stabilization method is supported by related studies by Yoshikawa and co‐workers, who used dicationic *N*,*N*‐dimethylbiguanidinium (DBG) to access the crystalline compound [DBG]_3_[V_10_O_28_].^[69]^ The group incorporated the material in LIB cathodes and reported a discharge capacity of 156 mA h g^−1^ in a voltage range between 1.5 and 3.8 V vs. Li^+^/Li. Further work by Liu and co‐workers showed that the combination of Mg^2+^ and NH_4_^+^ cations in Mg_2_(NH_4_)_2_[V_10_O_28_] also results in stable crystal lattices and gives access to LIB cathodes with discharge capacities of about 200 mA h g^−1^ in the potential range of 1.0 and 3.8 V vs. Li^+^/Li.^[70]^

These reports highlight the urgent need for fundamental studies on the reactivity of POVs, with a focus on thermally or chemically harsh conditions as used in many energy technologies. Based on this understanding, new concepts for stabilizing POVs (and POMs in general) could open new approaches for the design of materials for the field. In addition, fundamental questions also remain with respect to interfacial reactivity and the role of the nano‐ and microstructuring of POM‐based electrodes,^[16]^ particularly for applications where contact with electrolytes is essential. Pioneering works which address these questions will be discussed as part of the Outlook (Section 6).

## 4 POVs in Redox‐Flow Batteries

**Figure 4:**
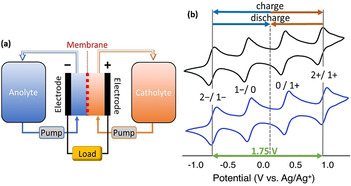
a) Diagram showing the typical setup and main components of an RFB. b) Voltammograms of [V_6_O_7_(OMe)_12_] (black) and [V_6_O_7_(OEt)_12_] (blue; for structures see Figure 1), showing possible charging/discharging processes. Adapted from Ref. [39]. Published by The Royal Society of Chemistry.

Redox‐flow batteries (RFBs) charge and discharge (highly concentrated) solutions of redox‐active species. The charged solutions can be stored in external tanks, which can be varied in size to allow inexpensive and simple scaling of the battery capacity without altering the electrode area. This results in decoupled power and capacity, which is not possible with other battery technologies and is an advantage for stationary energy‐storage applications.^[3]^ RFBs based on vanadate salts dissolved in aqueous acid have been successfully commercialized (Figure 4). However, their volumetric energy density is limited to around 50 Wh L^−1^ by the solubility of the vanadate salts.^[71]^ For comparison, Panasonic LIBs used in the 2013–2017 Tesla electric vehicles had energy densities of 670–683 Wh L^−1^.^[72]^ Intensive research is underway to develop next‐generation RFB technologies by creating new redox‐active materials to achieve higher energy densities and compete with LIB technology. This would enable RFBs to store more energy and operate on longer timescales, which is crucial for switching to renewable power sources. Improved specific and volumetric energy density would also allow RFBs to compete with LIBs in terms of applications in electric vehicles.

When designing new redox‐active species for RFBs, the two main chemical considerations are 1) how much charge can be stored per liter (volumetric capacity) and 2) how much energy can be stored per charge (the difference between the redox potentials of the redox couples in the separate electrolytes). Since RFBs are mainly aimed at (large‐scale) stationary energy storage, low materials cost is an important third consideration. Molecular and macromolecular species with multielectron storage capability (such as POMs or redox‐active polymers) can fulfil all three design criteria.[^73^, ^74^, ^75^, ^76^, ^77^, ^78^, ^79^] In one ground‐breaking study, a RFB based on a polyoxotungstate anolyte and a bromide/bromine‐based catholyte was reported to reach 200 Wh L^−1^,^[74]^ which is the energy density of the LIBs used in the 2013 Honda Fit electric vehicle.^[72]^

Although most of the POM‐RFB studies have thus far been focused on tungstate POMs, POVs offer some features which make them ideal charge‐storage materials in RFBs. POVs can often be accessed as mixed‐valence species, so that they can be both oxidized and reduced.^[23]^ This allows them to be used in symmetric RFBs, where the catholyte and anolyte use the same redox‐active species.^[73]^ POVs also have significantly lower molecular weights compared with molybdate and tungstate species. In the future, this should allow POVs to achieve higher specific energy densities and lower energy losses through electrolyte pumping compared to other heavier POM species.

In one key example, Stimming and co‐workers used a tungstate POM (H_4_[SiW_12_O_40_]) anolyte with a POV (Na_4.75_H_4.25_[PV^V^_14_O_42_]) catholyte.^[76]^ POMs/POVs generally exhibit electrochemically reversible (fast) electron transfer, while electron transfer in conventional all‐vanadium RFBs is typically slow. This limits the achievable current density and power output of typical RFBs. Stimming and co‐workers used the POM/POV system to achieve a power output that was 50 mW cm^−2^ greater than an all‐vanadium RFB.^[76]^ Prior to charging the RFB, [PV^V^_14_O_42_]^9−^ was chemically reduced to [PV^V^_10_V^IV^_4_O_42_]^13−^, which could then be oxidized by 4 electrons when charging the RFB catholyte. During charging, the H_4_[SiW_12_O_40_] anolyte was reduced by 2 electrons per cluster. Therefore, the clusters were used in a 2:1 molar ratio (tungstate/vanadate) to ensure that all the redox‐active material could be charged.^[80]^ This also illustrates the advantages of using two independent electrolytes in asymmetric RFBs, as the anolyte and catholyte can be adjusted individually to achieve an optimized whole‐cell performance. In addition, Stimming and co‐workers observed by ^51^V NMR spectroscopy that, upon degradation, the [PV_14_O_42_]^9−^ cluster reassembled under operational conditions. This behavior could lead to significantly longer operational lifetimes for POM‐based RFBs compared to other RFB systems.[^76^, ^81^] The POM/POV system has also recently been successfully scaled‐up and operated in a pilot‐scale reactor.^[81]^ These studies demonstrate the scalability and advantages of aqueous POM/POV RFBs.

In their efforts to increase the energy density, the RFB community has started to explore non‐aqueous RFBs. Switching to organic electrolytes increases the solubility of many redox‐active organic materials, and extends the functional voltage window of RFBs by preventing the electrolysis of water into oxygen and hydrogen.[^82^, ^83^] In a pioneering series of studies, Matson and co‐workers developed vanadium‐based Lindqvist clusters featuring alkoxy ligands for symmetric non‐aqueous RFBs. The highly organo‐soluble species [V^V^_2_V^IV^_4_O_7_(OR)_12_] (R=Me, Bu, C_2_H_4_OCH_3_, etc.) exhibited solubilities of up to 1.2 m in acetonitrile (containing 0.1 m *n*Bu_4_NPF_6_).[^29^, ^39^, ^84^, ^85^] The mixed oxidation states of the vanadium centers in [V^V^_2_V^IV^_4_O_7_(OR)_12_] allows the cluster to be reduced or oxidized by up to 2 electrons. This enabled the development of an RFB, where the same POV is used as the active species in the anolyte and catholyte. This can be advantageous because it prevents long‐term performance loss arising from active species crossover. Matson and co‐workers went on to demonstrate that chemical modification of their Lindqvist cluster can be used to tune the electrochemical performance. For example, tuning the redox potentials by incorporation of heterometals into the cluster framework is possible,^[29]^ whereas modification of the alkoxide ligands allows the electron‐transfer kinetics and the solubility (and therefore charge‐storage capacity) of the electrolyte to be tuned.[^29^, ^84^, ^85^, ^86^]

## 5 POVs in Light‐Driven Catalysis

The ability of POVs to absorb in the visible‐light region makes them well‐suited for photoredox catalysis using sunlight.^[87]^ Although traditional photoredox research on POMs was focused on UV‐light‐driven conversions of organic substrate by polyoxotungstates,^[88]^ the last decade has seen significant progress in POV‐mediated visible‐light‐driven photoredox processes.

**Figure 5:**
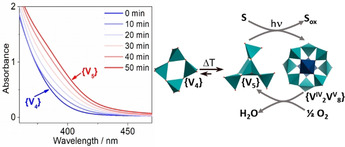
Left: Thermally induced structural rearrangement of **{V_4_}** to **{V_5_}**, and the resulting bathochromic shift of the light absorption. Right: Illustration of the light‐driven catalytic substrate oxidation cycle by **{V_5_}** (S=MeOH etc.; S_ox_=CH_2_O etc.).^[42]^

Early studies in visible‐light‐induced POV photooxidation chemistry explored the unique structural flexibility of the compound class to allow the in situ formation of visible‐light‐absorbing species. To this end, Streb and co‐workers used the thermal conversion of the UV‐absorbing cluster [V_4_O_12_]^4−^ (**{V_4_}**) to give the visible‐light‐absorbing [V_5_O_14_]^3−^ (**{V_5_}**).^[42]^ Irradiation of this species in organic solution with visible light in the presence of methanol resulted in the two‐electron/two proton oxidation of the alcohol to formaldehyde. Over the course of the reaction, the cluster underwent a reduction‐induced “dimerization” leading to the two‐electron‐reduced species [V^IV^_2_V^V^_8_O_26_]^4−^ (**{V^IV^**_**2**_ **V^V^**_**8**_ **}**). The authors were able to close the catalytic cycle by reoxidation of the decanuclear species using molecular oxygen (slow) or hydrogen peroxide (fast; Figure 5).

Subsequent studies showed that the photooxidative reactivity of POVs can be further tuned by the incorporation of high‐valent metal cations, for example, Ce^III[89]^ or Bi^III^.^[90]^ Notably, these systems sometimes even resulted in cerium‐ or bismuth‐functionalized POVs with helical chirality.[^91^, ^92^] Although initial studies only explored the photooxidative activity of the racemic mixtures of the respective POVs, future studies could build on this and target enantioselective photooxidation using the enantiopure cluster species.^[93]^

Building on these studies of metal‐functionalized POV photooxidation catalysts, Guldi, Streb, and co‐workers demonstrated that modification of the internal cluster template anion can also be used to modulate the photoreactivity. The authors explored the model compounds [*X*(Bi(dmso)_3_)_2_V_12_O_33_]^−^, which only differ in their internal template, where *X* is either chloride or bromide. Consequently, the two clusters show almost identical light absorption. The authors examined the photoredox activity of both clusters under identical conditions, using the photooxidative degradation of the model pollutant patent blue V (PBV) as a test reaction. This comparative study showed that the bromide‐templated system exhibited significantly faster PBV degradation kinetics compared with the chloride species. By using ultrafast photophysical analyses together with theoretical computations, it was proposed that, upon photoexcitation, the heavier bromide template enables a more efficient singlet–triplet transition because of the enhanced spin–orbit coupling (the so‐called heavy‐atom effect).^[91]^ This results in a faster reaction with the pollutant and the observed higher reaction kinetics. The same trend was observed for the quantum efficiencies of the dye degradation, which were significantly higher for the bromide‐templated species compared with the chloride‐based cluster. Many POV (and POM in general) photooxidation studies currently use molecular oxygen as a reoxidant to close the catalytic cycle. However, recent studies have shown that interfacial mass transport of O_2_ from the gas to the liquid phase can severely limit the catalytic performance of POVs.^[94]^ This finding emphasizes that detailed understanding is required across multiple scales to understand the optimization potential for these complex reactions, so that photophysical processes on the femtosecond can be effectively coupled to mass transport on the second timescale, and molecular design on the sub‐nanometer scale can be integrated with chemical reactor design on the micrometer scale and beyond.

The fields of light‐driven POV catalysis and POV metal functionalization have recently been combined to give access to bio‐inspired POV‐based water oxidation catalysts. In one example, Streb and co‐workers reported the manganese‐functionalized POV [Mn_4_V_4_O_17_(OAc)_3_]^3−^ (**{Mn_4_V_4_}**) as a functional model of the oxygen‐evolving complex [CaMn_4_O_5_], which enables water oxidation in the natural photosystem II.^[95]^ When coupled with the photosensitizer [Ru(bpy)_3_]^2+^ (bpy=2,2′‐bipyridine) and the terminal oxidant persulfate, the catalyst shows water oxidation under irradiation with visible light.^[95]^ Turnover numbers of about 12 000 and turnover frequencies of about 100 min^−1^ were achieved with this system.^[96]^ This first example of a POV water oxidation catalyst highlights the usefulness of stabilizing polynuclear, highly redox‐active transition‐metal‐oxo clusters as electron‐transfer and storage sites for (proton‐coupled) multielectron transfer. The example also highlights the vast opportunities offered by POMs for undertaking fundamental as well as applied water oxidation reactivity studies.^[97]^ Recent reports, for example, by Hayashi and co‐workers show that there might be a whole family of Mn‐functionalized vanadates with redox‐catalytic activity waiting to be explored.^[98]^

## 6 Outlook and Emerging Topics

To‐date, POV energy research has mainly been focused on component design for batteries and photoredox catalysis. However, the development of advanced POVs together with a deeper understanding of their reactivity and stability could lead to accelerated advances in established fields and new applications in sustainable energy research. The following section highlights current research opportunities in the areas discussed in Sections 3–5, as well as pioneering examples of emerging trends and future directions.

### 6.1 Future Directions in Established POV Energy Research

One major challenge in future POV energy research is a comprehensive analysis of POV reactivity and stability. This requires POV experts to collaborate closely with materials scientists, electrochemists, and photochemists to gain a deeper understanding of POV structure–function relationships. This could be used to develop new POVs with tunable reactivity and identify new POV structures as targets of future research. To date, most POV energy applications have been focused on a few POV prototypes, most notably [V_10_O_28_]^6−^, [PV_14_O_42_]^9−^, and [V_6_O_7_(OR)_12_]. This is a stark contrast to the fast‐paced recent progress in the design and functionalization of new POV architectures—many of which have not been studied for their possible use in energy technologies.[^21^, ^23^, ^27^, ^36^] Thus, new concepts such as POV modification with metal cations or organic ligands need to be aligned with the requirements of modern energy technologies. Ground‐breaking work in this direction has recently been reported by Matson and co‐workers, who used both organic functionalization and metal substitution in [V_6_O_7_(OR)_12_] to tune the redox reactivity and electron‐storage capability.[^29^, ^54^, ^99^] Related work by Streb and co‐workers showed that the incorporation of redox‐active transition metals into the organo‐soluble dodecavanadate [V_12_O_32_Cl]^5−^ is a facile route to tune redox behavior.^[100]^ More recently, the group showed that even incorporation of redox‐inactive Ca^2+^ ions into the dodecavanadate cluster leads to a significant enhancement of the cluster redox activity, thus allowing the fabrication of LIB cathodes with improved energy density. The authors attributed this surprising finding to the electrostatic and structural stabilization of the reduced cluster by Ca^2+^ ions.^[45]^

Beyond the chemical tuning of the POV itself, major challenges related to the interaction and deposition of POVs onto surfaces to construct electrodes still exist. These need to be overcome to establish general procedures for the stable “wiring” of POVs to electrodes or semiconductor surfaces. This is essential for any charge‐transfer application, and to prevent the leaching of POVs (e.g. in batteries or electrolysis), which is still a major challenge in the field. Recent initial studies have demonstrated that POV deposition on nanostructured carbon mediated by an ionic‐liquid “binder” could be one viable approach.^[101]^ In a related study, Sonoyama and co‐workers demonstrated that coating **{PV_14_}** nanoparticles with the conductive polymer (CP) polypyrrole results in significant improvements of the LIB capacity and cycling stability;^[102]^ thus, POM‐CP composites could open new research possibilities.^[103]^ In addition, organo‐functionalized POVs could be anchored to electrodes through covalent[^104^, ^105^] or supramolecular interactions.^[16]^ As an alternative approach, Walsh, Newton, Khlobystov, and co‐workers recently demonstrated the embedding of POMs inside electroactive carbon nanotubes. This ground‐breaking concept could in future be used to design composite electrode materials where the POM is electrically “wired” to a carbon support and leaching is virtually impossible.^[106]^

In contrast, the challenge for RFBs is how to keep POVs in different charge states in solution and prevent precipitation or decomposition. This is particularly challenging in organic electrolytes, even for organo‐functionalized POVs. Recent studies with tungstate POMs, however, have opened a facile route to overcome this issue. POM anions can be combined with large organic (often alkylammonium) cations to form so‐called POM ionic liquids (POM‐ILs), which are highly soluble in most organic solvents.[^107^, ^108^] We suggest that transferring this concept to POV chemistry could lead to highly organo‐soluble POV‐ILs for POV‐based RFBs. López and co‐workers have recently shown that computational methods can be used to understand and predict the behavior of POV/organocation mixtures in organic solvents, which could open new avenues towards designer RFB electrolytes.^[109]^

Another RFB‐related research theme requiring urgent attention is the effect of the supporting electrolyte on the POV performance. Very recently, Matson and co‐workers showed that replacing bulky, low‐charge‐density cations (e.g. *n*Bu_4_N^+^) in the supporting electrolyte with Li^+^ or Na^+^ significantly altered the redox potentials of their POV.[^29^, ^110^] The studies highlight that tuning the electrolyte composition in addition to tuning the POV is a promising means for controlling POV electrochemistry. That said, high‐enough concentrations of soluble POV salts or POV‐ILs would remove the need for added supporting electrolyte.

In the field of light‐driven POV chemistry, a major bottleneck is to increase the visible‐light harvesting of POVs, and new design concepts are required to shift the absorption of light by POVs (and POMs in general) into the visible region by tuning of the HOMO and LUMO gap. In addition, the ability to adjust the HOMO and LUMO energy levels (i.e. their redox potentials) would enable the targeted design of POVs for specific reduction or oxidation reactions. Inspiration for this HOMO–LUMO tuning might come from modern semiconductor chemistry, where modification of the analogous valence and conduction bands (e.g. by heteroelement doping) is well‐established.[^111^, ^112^] These concepts, however, have so far not been systematically transferred to POV chemistry and have only recently received attention for POMs in general.[^113^, ^114^, ^115^] In pioneering studies, Newton and co‐workers have demonstrated that the organic functionalization of Dawson polyoxotungstates can be used to control the HOMO–LUMO gap and HOMO–LUMO position, thereby enabling the controlled tuning of the resulting electro‐ and photochemistry.[^116^, ^117^]

An alternative approach towards higher visible‐light photoactivity is the coupling of POVs with metal complexes or organic photosensitizers,^[87]^ or POV deposition on light‐absorbing semiconductors.[^118^, ^119^] Principal studies in this direction have been undertaken with POMs; however, these studies also identified new challenges which arise from these more complex systems.^[118]^ In a landmark study, Kraus, Bren, Matson, and co‐workers have very recently expanded this concept to POVs: the authors deposited [V_6_O_7_(OEt)_12_] as hole scavengers on semiconducting CdSe light absorbers and demonstrated that this system shows enhanced light‐driven hydrogen evolution. The authors assign this observation to the ability of the POV to act as a hole scavenger, which facilitates proton reduction on the semiconductor surface.^[120]^

In the field of C−H activation, MacMillan and co‐workers recently reported a ground‐breaking study in which the tungstate archetype [W_10_O_32_]^4−^ was used as a photocatalyst to trigger the abstraction of a C−H hydrogen atom, which enabled the arylation^[121]^ or trifluoromethylation^[122]^ of aliphatic C−H bonds. This opens new opportunities for POV chemistry, where HOMO–LUMO tuning could be used to control reactivity and adjust selectivity as well as lead to control over product formation and the suppression of side reactions.^[87]^

### 6.2 Emerging Themes in POV Energy Research

POV battery research is now well‐established for LIBs and—to a lesser degree—NIBs. In contrast, other batteries, for example, Mg‐ or Ca‐based systems, have not yet been explored.[^123^, ^124^]

In addition, the use of POVs in supercapacitors has thus far not been explored in detail. Supercapacitors can store energy through a combination of electrical double layer capacitance and pseudocapacitance (i.e. faradaic electrochemical reactions),^[49]^ so the use of highly redox‐active POVs in supercapacitors seems promising. Ground‐breaking studies by Stimming, Srinivasan, and co‐workers reported the successful use of Na_6_[V_10_O_28_] as an electrode material for a supercapacitor,^[125]^ which gave an energy density of 73 Wh kg^−1^ and a power density of 312 W kg^−1^, based on double‐layer and pseudocapacitance. The study lays the foundations to expand POV research into the supercapacitor domain.

Another area which holds great potential for POVs is electrocatalysis. Here, POVs could be used as molecular species in homogeneous solution[^20^, ^97^, ^126^] or deposited on electrodes for heterogeneous applications.[^16^, ^103^, ^118^] To date, POV electrocatalysis is largely unexplored. Most of the existing studies reported the incorporation of POVs as a solid into carbon paste electrodes, so that analyses of the reactivity and stability of the POV under electrochemical conditions is challenging.^[62]^ In addition, the studies often targeted facile electrocatalytic processes, for example, the conversion of nitrite, bromate, iodate, or ascorbic acid.[^127^, ^128^, ^129^] In contrast, little has been reported on more challenging (proton‐coupled) multielectron reactions for small‐molecule activation (e.g. H_2_, O_2_, CO_2_, N_2_). In one recent example, Streb and co‐workers demonstrated that the molecular‐water oxidation catalyst **{Mn_4_V_4_}** (see Section 4) also shows sustained oxygen evolution under electrochemical conditions;[^95^, ^97^] however, the study provided little insight into the mechanism for the oxidation of water, and no information is available on the cluster stability or the role of the vanadate ligand during catalysis. In another pioneering example, Li, Yang et al. used Cu^+^ ions to link organo‐functionalized Lindqvist POVs into metal–organic frameworks, which showed intriguing reactivity for the electrocatalytic oxygen reduction reaction when deposited on a carbon electrode.^[130]^ Building on these pioneering reports, future studies could focus on exploring structure–reactivity–stability relationships on the molecular level to identify the nature of the electrocatalytically active species. In addition, focus on technologically important reactions is required so that fundamental mechanistic studies and application‐driven POV development can be combined to access high‐performance electrocatalysts. Again, the stable “wiring” of POVs to electrode surfaces is still virtually unexplored and forms a prerequisite to access systems with high durability and technological relevance.^[118]^

In the field of small‐molecule activation, Kikukawa et al. have recently broken new ground by using the bowl‐shaped dodecavanadate [V_12_O_32_]^4−^ to bind elemental Br_2_ in the central cavity, thereby leading to polarization of the Br−Br bond and significant selectivity changes in organic bromination reactions.^[131]^ In a related example, Das and co‐workers demonstrated that POVs can sequester CO_2_ from air and store it as an internal CO_3_^2−^ template, thereby resulting in the species [H_8_V^IV^_15_O_36_(CO_3_)]^6−^.^[132]^ If this approach can be coupled to a reversible template release (as reported for several POMs),[^133^, ^134^, ^135^] this discovery could open new horizons for the capture and storage of molecular carbon using the unique structural disassembly and reassembly properties of polyoxometalates.[^32^, ^40^, ^43^, ^136^, ^137^]

## 7 Conclusion

POVs have proven to be versatile redox‐active materials that can be used to overcome challenges in many sustainable energy applications, from batteries to light‐driven catalysis. Despite this, only a small fraction of the known POV cluster types have been investigated as components of energy conversion or storage systems. Furthermore, more extensive investigation of the physical properties of existing species is needed to use them effectively in energy applications, such as, determining trends in redox behavior, photophysical properties, thermal stability, and solubility. Expanding the available knowledge of POV properties will allow the field to move beyond the model POVs described here and allow POVs to be designed to overcome current limitations. By addressing the issues we have highlighted, we believe that POVs can become key components in the development of future sustainable energy storage systems.

## Conflict of interest

The authors declare no conflict of interest.

## Biographical Information

*Montaha Anjass is an independent group leader and Margarete‐von‐Wrangell‐Fellow at Ulm University and Helmholtz Institute Ulm. She received her B.Sc. in Chemistry from Birzeit University*, *Palestine, and her M.Sc. in Advanced Materials from Ulm University, Germany. In 2019, she completed her PhD (supervisors: C. Streb, M. Fichtner, T. Jacob) at Ulm University. Her current research interests are advanced battery materials based on redox‐active molecular metal oxides and electrically conductive organic polymers, and their use in (post‐)lithium batteries*.

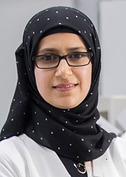

## Biographical Information

*Grace A. Lowe is a postdoctoral researcher in the Streb group at Ulm University. She completed her PhD at the Centre for Doctoral Training in Sustainable Chemistry at Nottingham University in 2019 under the supervision of Dr. D. A. Walsh and Dr. G. N. Newton. Her current research interests are redox‐flow batteries and the electrochemical characterization of inorganic materials, mainly, but not limited to, POMs*.

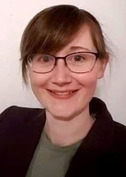

## Biographical Information

*Carsten Streb is Professor of Inorganic Chemistry at Ulm University and group leader at the Helmholtz‐Institute Ulm. His love for POMs started during his PhD, where he worked with Lee Cronin at the University of Glasgow. His current research is focused on designing POM‐based functional materials and composites to address global chemical challenges, including energy conversion/storage, water purification, and public health*.